# CGAP: a new comprehensive platform for the comparative analysis of chloroplast genomes

**DOI:** 10.1186/1471-2105-14-95

**Published:** 2013-03-14

**Authors:** Jinkui Cheng, Xu Zeng, Guomin Ren, Zhihua Liu

**Affiliations:** 1 Department of Computational Biology and Bioinformatics, Institute of Medicinal Plant Development, Chinese Academy of Medical Sciences & Peking Union Medical College, Beijing, 100193, China; 2 Nanjing Forestry University, Nanjing, 210037, China

**Keywords:** Chloroplast genomes, Comparative and phylogenetic analysis, Web-based platform

## Abstract

**Background:**

Chloroplast is an essential organelle in plants which contains independent genome. Chloroplast genomes have been widely used for plant phylogenetic inference recently. The number of complete chloroplast genomes increases rapidly with the development of various genome sequencing projects. However, no comprehensive platform or tool has been developed for the comparative and phylogenetic analysis of chloroplast genomes. Thus, we constructed a comprehensive platform for the comparative and phylogenetic analysis of complete chloroplast genomes which was named as chloroplast genome analysis platform (CGAP).

**Results:**

CGAP is an interactive web-based platform which was designed for the comparative analysis of complete chloroplast genomes. CGAP integrated genome collection, visualization, content comparison, phylogeny analysis and annotation functions together. CGAP implemented four web servers including creating complete and regional genome maps of high quality, comparing genome features, constructing phylogenetic trees using complete genome sequences, and annotating draft chloroplast genomes submitted by users.

**Conclusions:**

Both CGAP and source code are available at http://www.herbbol.org:8000/chloroplast. CGAP will facilitate the collection, visualization, comparison and annotation of complete chloroplast genomes. Users can customize the comparative and phylogenetic analysis using their own unpublished chloroplast genomes.

## Background

The chloroplast is an essential organelle in plants which performs photosynthesis. Chloroplast contains independent genome derived from a cyanobacterial ancestor [1]. Chloroplast genome typically consists of circular double-stranded DNA molecules of 110–200 kb size, including 100–200 unique genes. Most chloroplast genomes contain two large inverted repeats (IRs) of 6–76 kb which are highly conserved and divide the genomes into one large and one small single-copy region (called LSC and SSC, respectively) [2]. The chloroplast genomes contain important genes involved in photosystems and biosynthetic pathways. Many coding and non-coding sequences of chloroplast genomes have been used for the phylogeny analysis of plants, including: *rbcL, matK* and *psbA-trnH*[3,4]. Because of the conserved nature, appropriate size, persistent gene organization and potential ability for plant phylogenetic inference and transgenic expression, chloroplast genomes have been widely sequenced and used for the comparison and phylogeny analysis [5-7].

As the number of sequenced chloroplast genomes increases rapidly, bioinformatics tools become more critical for the analysis of complete chloroplast genomes. GenBank, EMBL and DDBJ are the primary nucleotide sequences databases. The chloroplast genome database (CGDB: http://nar.oxfordjournals.org/content/34/suppl_1/D692.full) and GOBASE (http://gobase.bcm.umontreal.ca/) are specialized chloroplast repositories [8,9]. Dual organelle genome annotator (DOGMA: http://dogma.ccbb.utexas.edu/) is a web-based annotation tool for chloroplast and mitochondrial genomes [10]. GeneOrder (http://binf.gmu.edu:8080/GeneOrder3.0/) and BADGER (http://badger.duq.edu/manual2/models.html) can be used for comparative analysis of gene arrangements in small genomes [11]. GRAPPA (http://www.cs.unm.edu/~moret/GRAPPA/) and MGR (http://grimm.ucsd.edu/MGR/) perform phylogenetic analysis based on gene order changes [12-14]. Several tools offer the option to create chloroplast genome maps (e.g. PlasMapper, CGView and OGDRAW) [15-17]. However, there is no comprehensive platform or tool which can be used for the comparative and phylogenetic analysis of chloroplast genomes. We aim to construct a platform which integrates genome collection, visualization, comparison, phylogenetic analysis and annotation functions together. It will facilitate the comparative and phylogenetic analysis of complete chloroplast genomes.

## Implementation

**Figure 1:**
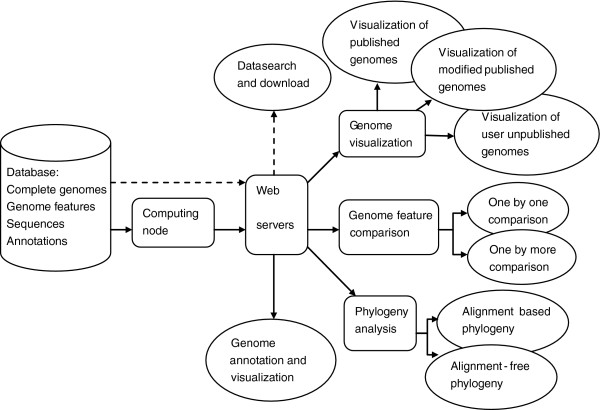
Architecture of CGAP.

CGAP contains a built-in database and four web servers including visualization of genomes, comparison of genome features, phylogeny analysis and genome annotation. The architecture of the platform was showed in Figure 1. CGAP was implemented using Python programming language and Web2py web framework (http://www.web2py.com). Entire platform was constructed on a machine with 16 GB RAM. The performances of the database and web servers were tested via a variety of web browsers (e.g. IE, Firefox, Chrome and Safari). As of writing this article, CGAP has been running for half a year.

## Results and discussion

CGAP collected 284 complete chloroplast genomes from NCBI Organelle Genome Resources (http://www.ncbi.nlm.nih.gov/genomes). According to the annotation information stored in the GenBank format file, CGAP extracted all types of genome features including Gene, CDS, tRNA, rRNA, Exon, Intron, Promoter, RepeatRegion, StemLoop, -10 Signal and -35 Signal. Complete chloroplast genomes and all genome features were stored in CGAP chloroplast database. You can view and download all genomes and features in Fasta format online.

### Visualization of genomes

**Figure 2:**
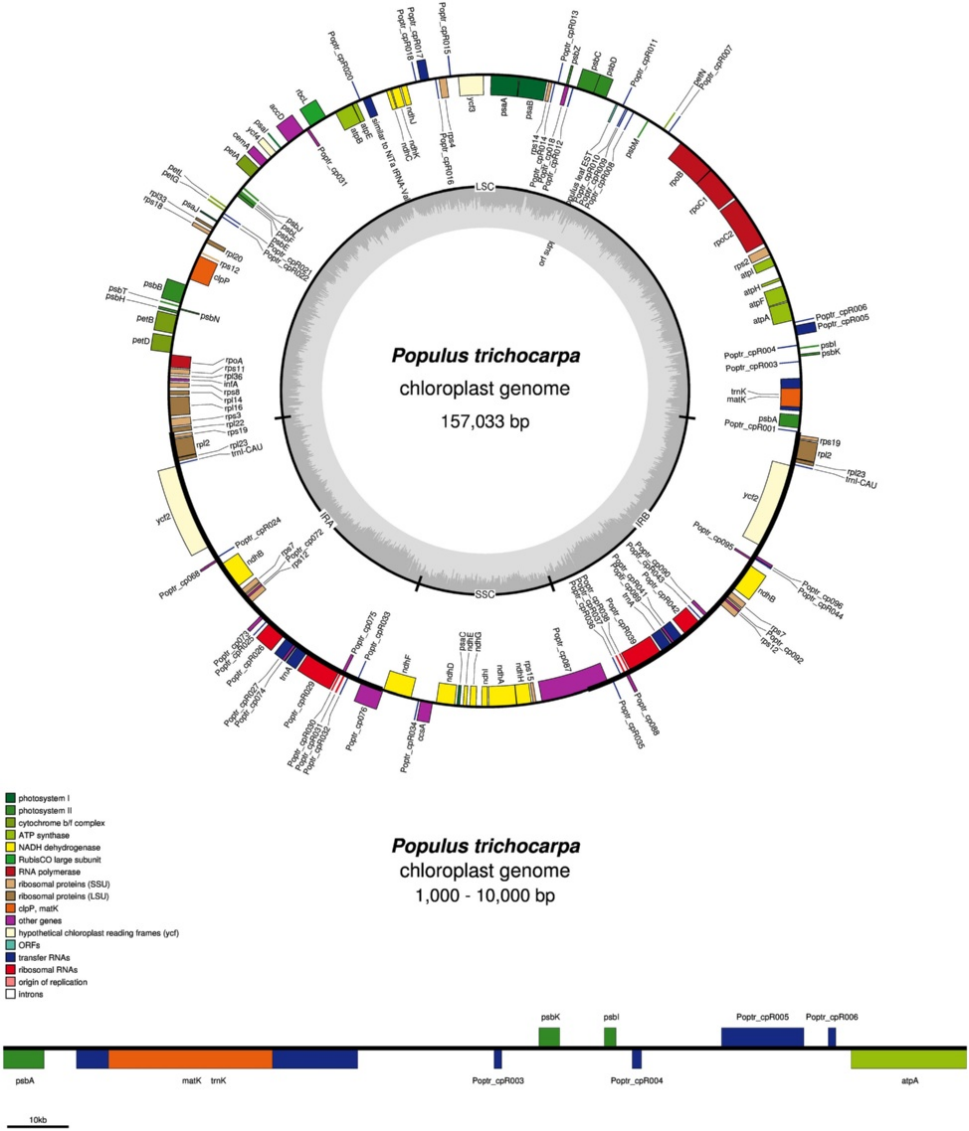
**Complete and regional genome map of *Populus trichocarpa.***

In order to better illustrate chloroplast genomes, CGAP implemented three functions for the visualization of genomes, including the visualization of circular complete genomes and linear regional genomes, the visualization of modified published genomes, and the visualization of user unpublished genomes. Complete and regional genome maps of *Populus trichocarpa* [GenBank: NC_009143.1] were showed in Figure 2; All functions used Perl modules (including BioPerl, PerlMagick, PostScriptSimple, TestSimple and PerlXML) and OGDRAW to create high quality genome maps [17]. In the genome maps, different features were indicated by different colors, and every feature was annotated using its name. For each genome map CGAP provided five types of figures for viewing and downloading, including TIFF, PNG, JPG, GIF and PS. In order to create maps of the modified published genome, user needs to indicate the genome using its organism name or accession number, and submit a file which contains the modified items of the published genome. Every line contains one modified item which has three fields separated by comma, including FeatureName, the Start and End position. For maps of unpublished genomes, user needs to submit the annotation file of the genome. The first part of the annotation file contains the annotation items, one annotation item per line. Every annotation item has four fields separated by comma, including FeatureType, FeatureName, the Start and End position. The second part of the annotation file contains the complete genome sequence in Fasta format. Model files for test can be found from the website where it is used.

### Comparison of genome features

**Figure 3:**
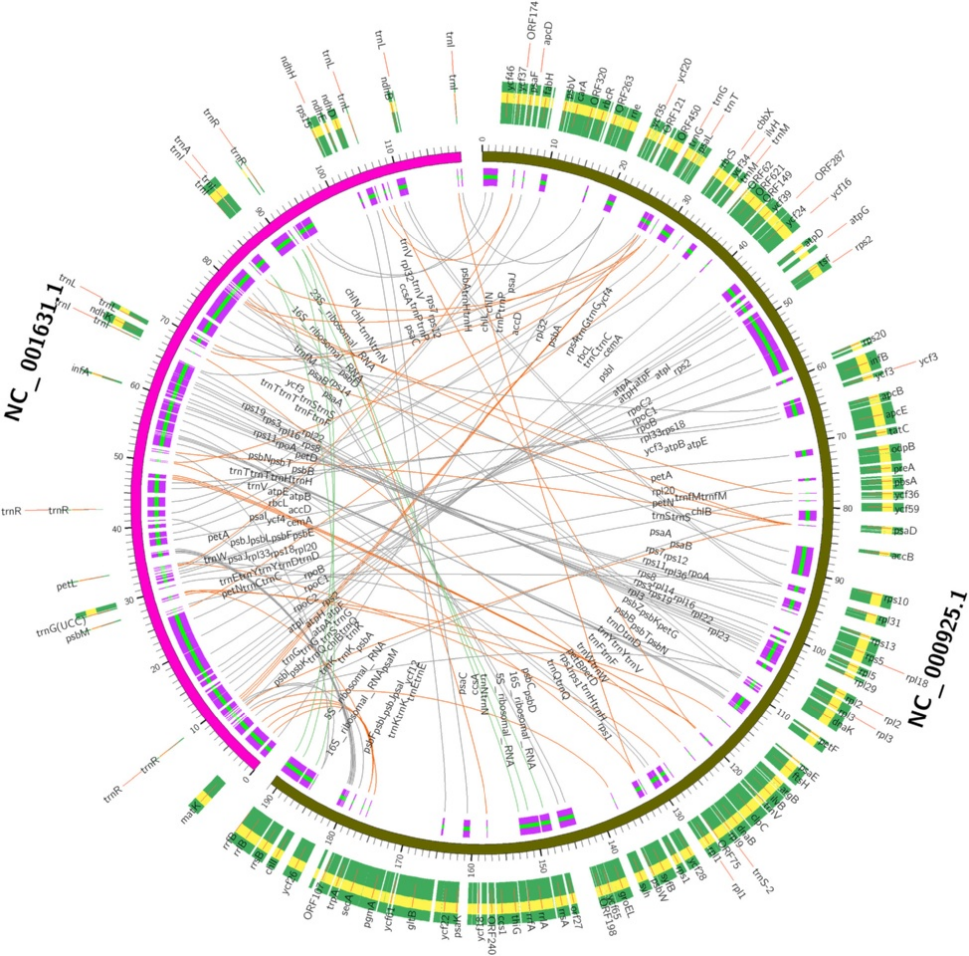
**Feature content comparison results between *Pinus thunbergii *and *Porphyra purpurea.***

**Figure 4:**
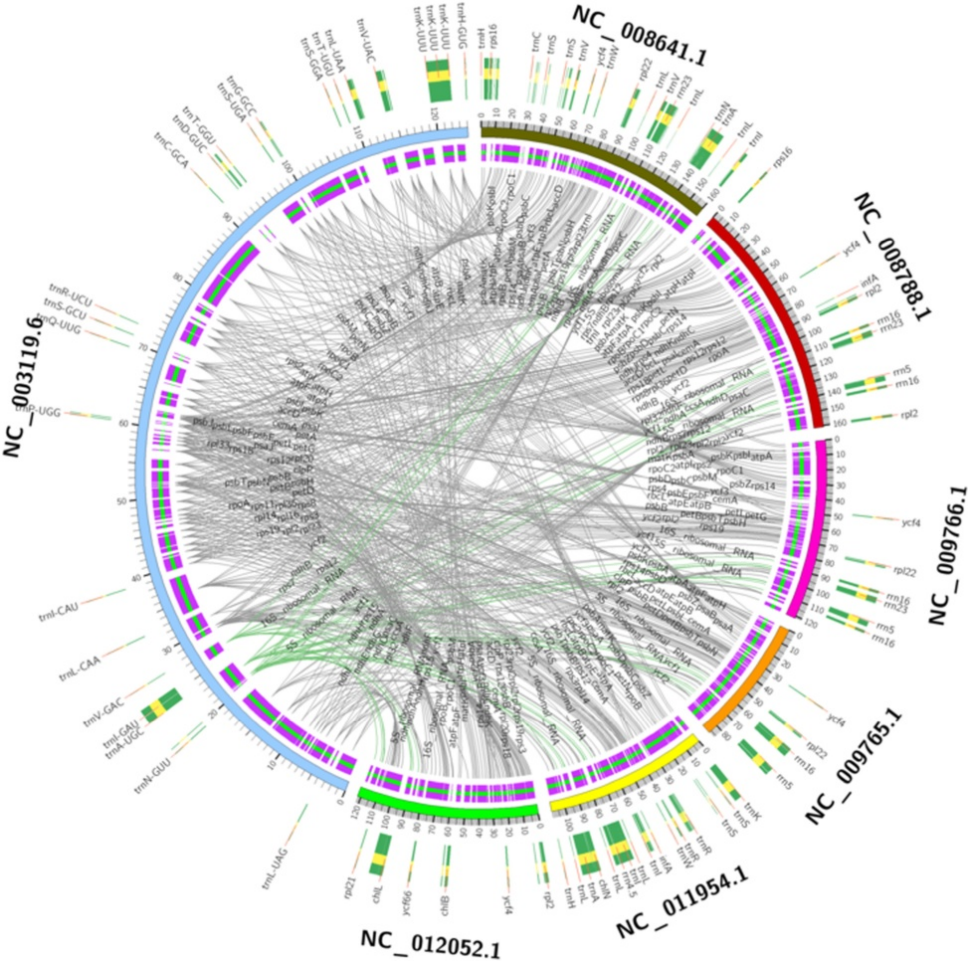
One by more comparison results of feature content.

The feature content of chloroplast genome gives detailed information about the composition of the genome. In general, chloroplast genomes differ from each other in feature content. CGAP compared the similarities and differences of the feature content between different genomes, which was implemented based on text mining method and the annotated feature information of the genomes. CGAP also visualized the comparison results in high quality, detailed circular layout using Circos [18]. CGAP implemented two functions for the comparison of feature content, including one by one and one by more comparison. Figure 3 showed the comparison results between *Pinus thunbergii* [GenBank: NC_001631.1] and *Porphyra purpurea* [GenBank: NC_000925.1]. Figure 4 showed the comparison results of *Medicago truncatula* [GenBank: NC_003119.6] versus *Gossypium barbadense* [GenBank: NC_008641.1], *Nuphar advena* [GenBank: NC_008788.1], *Cuscuta reflexa* [GenBank: NC_009766.1], *Cuscuta gronovii* [GenBank: NC_009765.1], *Ephedra equisetina* [GenBank: NC_011954.1] and *Syntrichia ruralis* [GenBank: NC_012052.1]. In both Figures the internal annotations and lines between genome features indicated the same features between the genomes compared, and the external annotations indicated the different features. For both types of comparisons, you can submit your own draft genome and customize the chloroplast genomes used in your comparative analysis. For one by more comparison using only the published genomes, user needs to submit a file which contains the names of organisms or accession numbers of the genomes compared. All names are placed in the first line of the file and separated by comma. For one by more comparison using the user unpublished genome, all names are also placed in the first line and separated by comma, and the first name indicates the user unpublished genome. The second line describes the length of user genome. From the third line to the end of the file, each line contains one focused feature of the user genome. Optionally, you can supply a range of the genome, and then CGAP will compare the genomes and visualize the comparison results only on the focused range of the genome. Results of the regional comparison of genomes can be seen in Additional files 1 and 2.

### Phylogeny analysis

Traditional phylogeny analysis is based on multiple sequence alignment. Sequence alignment methods meet huge challenge when dealing with large-scale complete genomes. Thus, various alignment-free methods have been proposed [19,20]. CGAP used a novel sequence feature called base-base correlation (BBC) to characterize the complete chloroplast genome. BBC was first proposed by Liu et al. [21,22]. For each chloroplast genome CGAP extracted one BBC feature vector, and then calculated the distance matrix of the feature vectors using one of the ten distance methods implemented in CGAP. Finally, CGAP constructed the phylogenetic tree based on the distance matrix and neighbor-joining (NJ) method [23]. In order to compare the results of alignment-free method with traditional alignment-based method, CGAP also implemented phylogenetic analysis based on whole genome sequence alignment. The alignment of whole genome sequences was performed using MUMmer, and the distance of genomes was calculated using following formula [24].

$$D_{\mathrm{AB}}=-\mathrm{lo}g_{2}\frac{N_{\mathrm{mat}}}{L_{\max}}$$

Where, *N*_*mat*_ denotes the number of nucleotides matched between genomes *A* and *B*, *L*_*max*_ is the max length of all genomes analyzed.

CGAP saved the distance matrix of the genomes as three kinds of formats, including the standard Nexus format and distance formats used in MEGA and PHYLIP [25,26]. CGAP also drew a tree map for the overview of the phylogenetic relationship (see Additional file 3), and saved the phylogeny tree as standard Newick and Nexus formats. Optionally, you can supply your unpublished genomes and customize the chloroplast genomes used in your phylogeny analysis. In this situation, users need to submit a txt file, the first part of the file contains all names of organisms or accession numbers of the published genomes used in the analysis process, and the second part of the file contains the unpublished complete genomes in Fasta format.

### Genome annotation

CGAP annotated new chloroplast genomes based on feature sequences of the chloroplast genomes collected in CGAP database and basic local alignment method (BLAST 2.2.25+: http://blast.ncbi.nlm.nih.gov/) [27]. CGAP identified the potential elements of your genome according to the sequence similarities between the elements and the features in the database. Then, CGAP attached biological information to the elements identified based on the information of the most similar feature [28-32]. Finally, CGAP returned you a list of non-redundant annotated entries which described the potential features on your genome. Every annotated entry for a segment sequence of your genome has 8 fields, including NormalizedFeatureName, Start, End, FeatureName or Location, LengthRatio, Identity, Score and Expectation. The meaning of each field was described in Table 1. CGAP also visualized the genome in high-quality circular map based on the annotations.

**Table 1:** Meanings of the fields in annotation entry

**Fields of annotation entry**	**Meanings of the fields**
Normalized FeatureName	Normalized feature name of a segment sequence of the genome
Start	Starting position of the segment sequence on the chloroplast genome
End	Ending position of the segment sequence on the chloroplast genome
FeatureName/Location	Potential feature name or location identifier of the segment sequence of the genome
LengthRatio	|End-Start|/L, L indicated the length of the feature sequence compared to the segment sequence of the genome
Identity	Nmatch/Nmissmatch, Nmatch and Nmissmatch indicated the number of match and mismatch bases of the segment sequence respectively in sequence alignment
Score	Score obtained in sequence alignment
Expectation	Expectation value computed in sequence alignment

## Conclusions

CGAP was developed for the comparative analysis of complete chloroplast genomes. It integrated genome collection, visualization, content comparison, phylogeny analysis and annotation functions together. CGAP implemented feature content comparison of chloroplast genomes and a novel alignment-free method for the phylogenetic analysis. Users can customize the comparative and phylogenetic analysis using their own unpublished genomes. To our knowledge, CGAP represents the first comprehensive platform for the comparative analysis of chloroplast genomes. It would facilitate the researches and applications of complete chloroplast genomes.

## Availability and requirements

**Project name**: CGAP

**Project home page**: http://www.herbbol.org:8000/chloroplast

**Operating system(s)**: Linux for the distributed source code and operating system independent for the web servers

**Programming language**: Python 2.6

**License**: Free for academic use

## Abbreviations

CGAP: Chloroplast genome analysis platform; IRs: Inverted repeats; LSC: Large single-copy; SSC: Small single-copy; BBC: Base-base correlation; NJ: Neighbor-joining

## Competing interests

The authors declare that they have no competing interests.

## Authors’ contributions

JC implemented the programs, and wrote the initial manuscript draft. XZ supplied constructive suggestions for the functions of the platform. GR set up the frame work for the web server. ZL designed the whole study, proposed the alignment-free method and revised the manuscript extensively. All authors read and approved the final manuscript.

## Supplementary Material

Additional file 1 One by one regional comparison results of genomes. Click here for file

Additional file 2 One by more regional comparison results of genomes. Click here for file

Additional file 3 Overview of the phylogenetic tree constructed in phylogeny analysis. Click here for file
