# Supplementary figures and images for: CGAP: a new comprehensive platform for the comparative analysis of chloroplast genomes
Source: BMC Bioinformatics. 2013 Mar 14;14:95. doi: 10.1186/1471-2105-14-95 (PMC3636126; doi:10.1186/1471-2105-14-95)

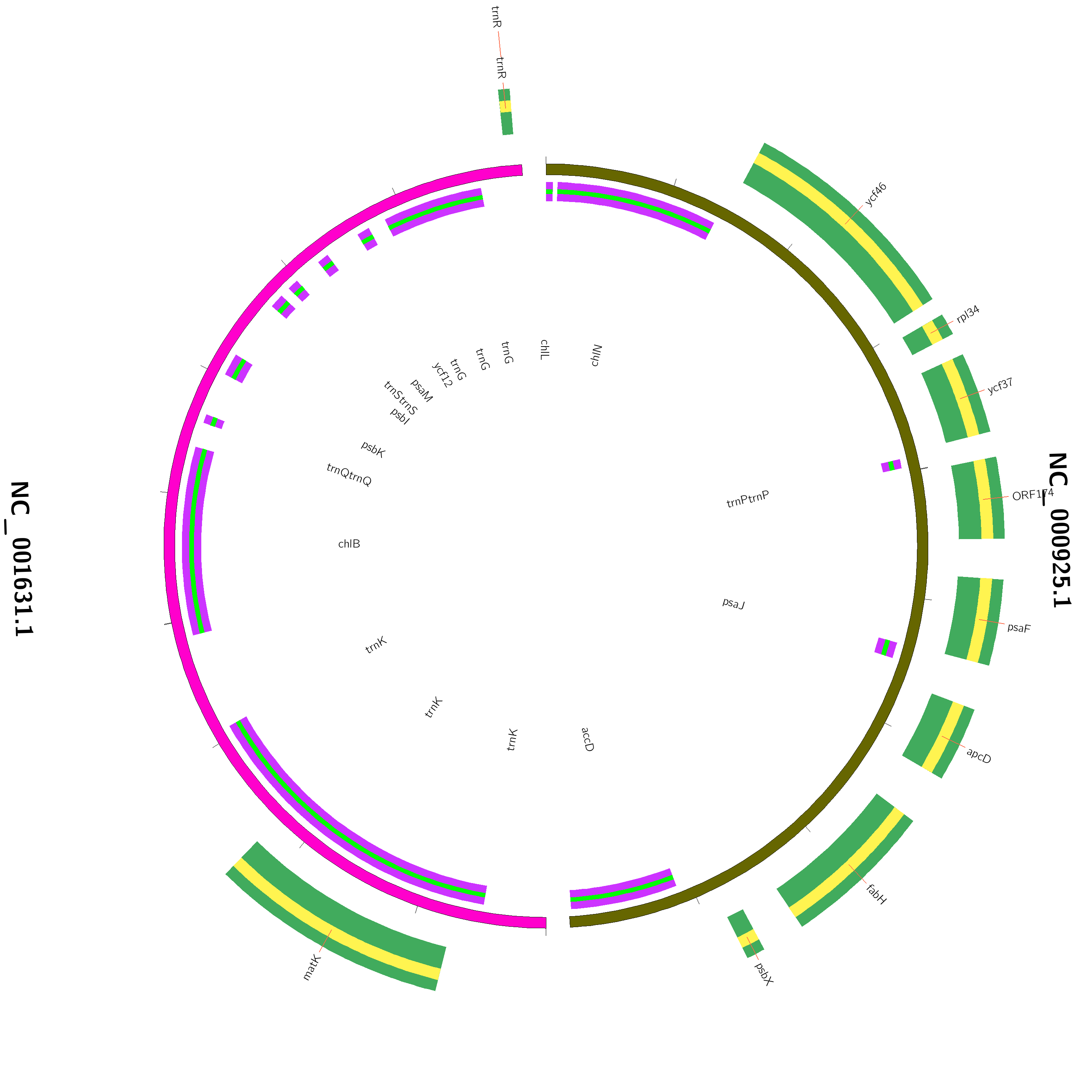

Supplement: Additional file 1 — One by one regional comparison results of genomes. [file 1471-2105-14-95-S1.png]

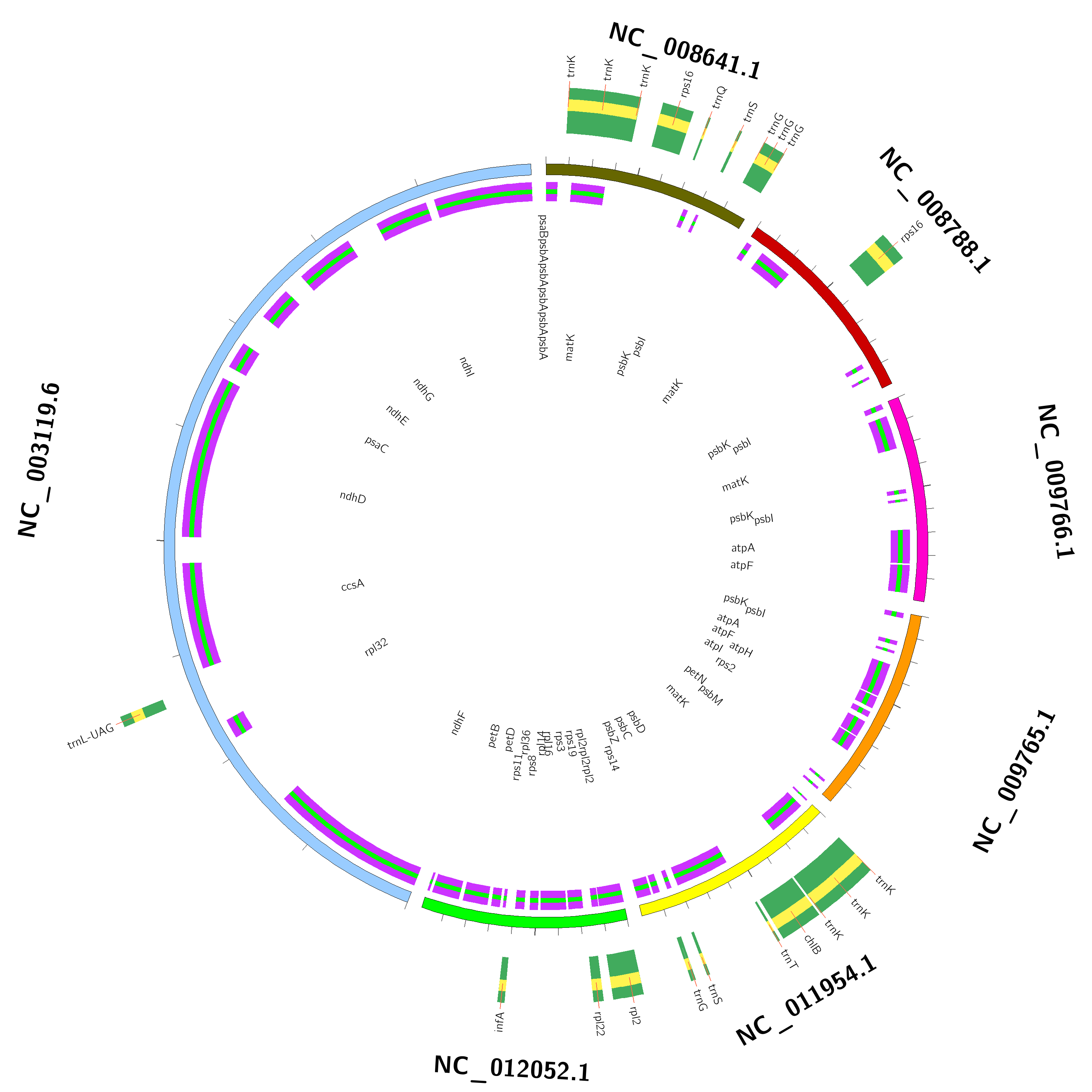

Supplement: Additional file 2 — One by more regional comparison results of genomes. [file 1471-2105-14-95-S2.png]

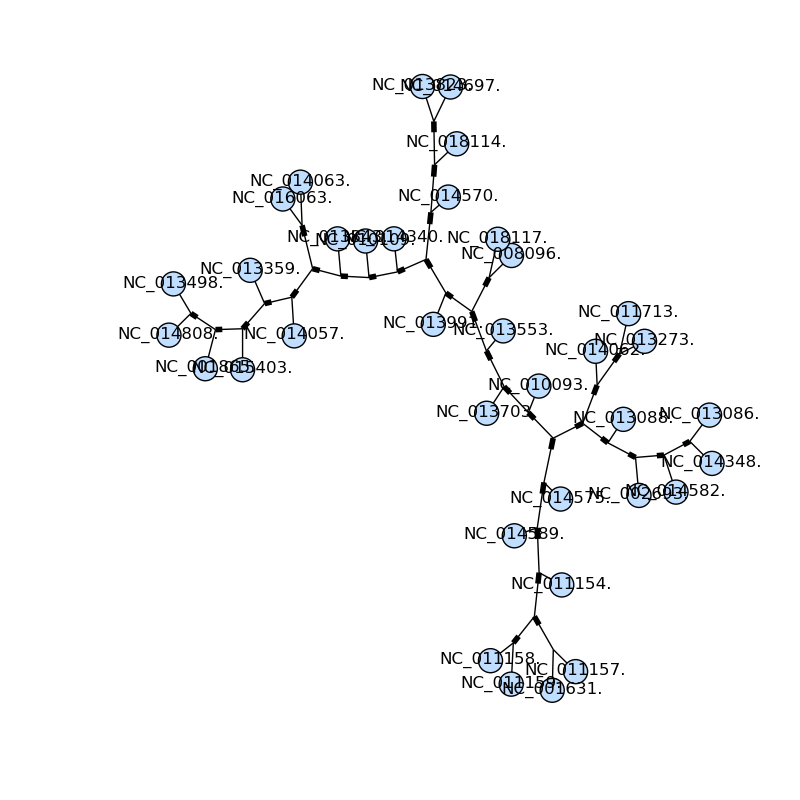

Supplement: Additional file 3 — Overview of the phylogenetic tree constructed in phylogeny analysis. [file 1471-2105-14-95-S3.png]
